# Failure Analysis and Thermo-Mechanical Simulation of Seal Welding and Girth Welding in Lined Composite Pipes

**DOI:** 10.3390/ma19132693

**Published:** 2026-06-23

**Authors:** Xianqiao Fu, Hai Fu, Yuanxin Jiang, Ze Wu, Yang Yu, Bin Han, Tianping Gu

**Affiliations:** 1 School of Materials Science and Engineering, China University of Petroleum (East China), Qingdao 266580, China; 2 China National Petroleum Engineering Construction Corp North China Branch, Renqiu 062522, China; 3 Xi’an Sunward Aeromat Co., Ltd., Xi’an 710025, China; 4 Northeast Sichuan Gas District of PetroChina Southwest Oil and Gas Field Company, Dazhou 635000, China; 5 School of Mechanical Engineering, Xi’an Shiyou University, Xi’an 710065, China

**Keywords:** lined pipe, weld overlay, girth welding, thermal history, residual stress

## Abstract

This study focused on burn-through leakage at girth welds of mechanically lined pipe (MLP) during field service. Field failure analysis, experimental tests, and numerical simulation were combined to investigate the process parameters of seal welding and multi-pass girth butt welding. Macroscopic metallography and energy dispersive spectroscopy (EDS) of failed specimens showed that excessive welding heat input (high current) caused severe expansion of the heat-affected zone (HAZ) and significant element dilution. The results indicated that the HAZ width of the solid-wire girth weld increased markedly from 1.312 mm to 2.247 mm under high-current conditions. Meanwhile, the Fe mass fraction in the root pass sharply increased to 33.66%, while key corrosion-resistant elements such as Cr and Ni were greatly reduced, which directly led to local pitting corrosion and perforation leakage. In addition, a moving heat source model was established in Abaqus 2024 to simulate the multi-pass welding process. The results showed that strong stress concentration developed at the groove root and the interface between the backing steel pipe and corrosion-resistant liner during repeated thermal cycles. The maximum von Mises stress reached 686.56 MPa during the second butt welding pass. After final cooling, the residual hoop tensile stress and axial tensile stress at the center of the inner surface reached 500–550 MPa and 480–510 MPa, respectively. By correlating microscopic compositional evolution with the macroscopic residual stress field, this study revealed the weld failure mechanism of MLP joints. The proposed finite element method can also be used as an efficient tool to predict the effects of welding speed, current, and voltage on residual stress, providing guidance for field welding procedure optimization and pipeline structural integrity assessment.

## 1. Introduction

Mechanically lined pipe (MLP) consists of an outer carbon steel backing pipe and an inner corrosion-resistant alloy (CRA) liner, combining pressure-bearing capacity with corrosion resistance. It has been widely used in oil and gas gathering and transportation pipelines under severe service environments containing high H_2_S and high chloride concentrations [1,2,3,4,5]. However, during field construction, the connection of MLP mainly relies on multi-pass girth welding. Due to the significant difference in material properties, the microstructural evolution and mechanical response in the butt joint region are highly complex, making it a weak point in pipeline systems.

**Figure 1:**
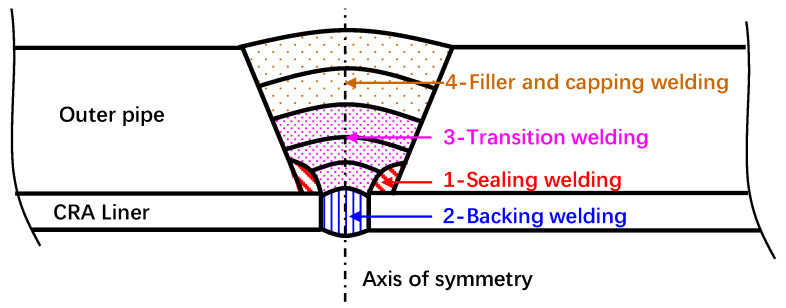
Schematic of butt welding for MLP.

As shown in Figure 1, MLP butt joining usually adopts a multi-layer and multi-pass welding procedure consisting of seal welding, root welding, hot pass welding, filling, and cap welding. During this process, the local joint region experiences repeated non-uniform thermal cycles, which not only cause significant element diffusion and dilution, but also lead to repeated accumulation of residual stress [6,7]. In some field cases, burn-through leakage defects after seal welding and subsequent girth welding were mainly concentrated near the fusion line and adjacent to the seal weld, showing obvious local corrosion and perforation features. This indicates that seal welding is not only a simple pre-assembly process, but its welding parameters and heat input may have a critical influence on the microstructural evolution and service performance of the subsequent welded joint. Therefore, as the initial step for connecting the liner and backing pipe [8], the heat input and forming condition of seal welding directly affect the heat transfer path and residual stress accumulation during the subsequent welding process [9,10,11].

In addition, due to the significant mismatch in thermal expansion coefficients and mechanical properties between the inner and outer layers, high tensile residual stress is likely to develop at the root of multi-pass girth welds [12], This stress concentration can directly reduce the fatigue life of the joint and promote stress corrosion cracking (SCC). For such complex material combinations, numerical simulation combined with experimental testing has confirmed that optimizing groove design and post-weld cooling paths can effectively adjust the location of stress peaks, thereby improving structural integrity [13,14,15]. At the same time, precise control of heat input is considered a key factor in ensuring weld quality. It not only controls microstructural and compositional evolution, but also indirectly affects residual stress distribution. Excessive welding heat input can significantly enlarge the geometric size of the HAZ and strongly accelerate the diffusion of Fe from the carbon steel backing pipe into the liner root weld metal [16]. This causes severe depletion of key corrosion-resistant alloying elements (Cr, Ni, and Mo) near the fusion line, thereby reducing passive film stability and increasing susceptibility to local corrosion [17]. In addition, the selection of filler materials has also been confirmed to influence the final microstructural evolution and stress distribution of the weld [18].

For seal welding, which is a unique and critical process in MLP fabrication, the welding parameters not only determine the initial stress state at the interface between the liner and backing pipe, but also directly affect the interlayer thermal resistance and stress superposition during subsequent butt welding. If the heat input of the seal weld is too high, local thermal instability and wrinkling of the liner may occur, creating potential risks for later burn-through leakage failure [19,20]. Although previous studies have achieved progress in individual welding processes or material effects [21,22,23], coupled investigations covering the full procedure of “seal welding + multi-pass butt welding” for MLP are still limited. On one hand, most existing numerical models neglect the influence of seal welding on subsequent thermal cycles and stress accumulation. On the other hand, the combined effect of microscopic element dilution and macroscopic residual stress has not been systematically quantified, making it difficult to fully explain the mechanism of early weld leakage in practical engineering applications.

Therefore, this study systematically investigated the perforation and leakage mechanisms of girth welds in composite pipes by combining on-site failure sample analysis with thermo-mechanical finite element simulations. Macroscopic metallographic observations and EDS analysis revealed the influence of welding heat input on elemental diffusion and distribution characteristics within the HAZ. Meanwhile, a finite element model encompassing the entire sealing and multi-pass girth welding process was established to obtain the evolution of the post-weld residual stress distribution. Based on these results, the weld failure mechanism was comprehensively elucidated from the perspectives of elemental dilution and local stress concentration, and the effects of key welding parameters on joint service performance were further evaluated. The findings provide a theoretical basis and methodological reference for optimizing on-site welding procedures and designing corrosion-resistant structures for composite pipes.

## 2. Failure Analysis of Failed Specimens

To investigate the actual cause of burn-through leakage in field MLP, relevant examinations were carried out on girth butt welds with pitting leakage defects. The main test items included macroscopic and microscopic metallographic analysis of the girth welds, as well as chemical composition analysis.

### 2.1. Metallographic Analysis

**Figure 2:**
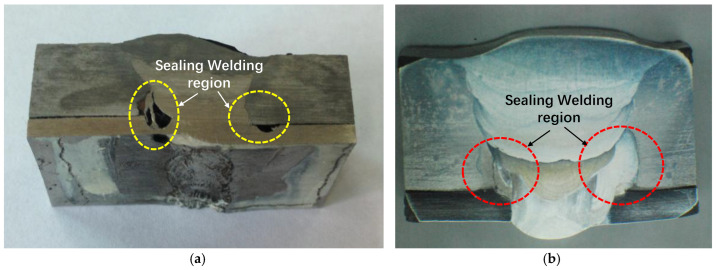
Macrostructure specimen of the girth butt weld. (**a**) Field burn-through leakage specimen; (**b**) macroscopic metallography of field specimen.

To determine the visual characteristics and defect locations of perforation failure in the composite pipe, three samples were extracted from the field, and the most representative girth butt-weld sample was selected for macroscopic metallographic observation, as shown in Figure 2. Figure 2a shows obvious corrosion perforation in the failed specimen. Although no deep pit was observed in Figure 2b, local rust marks had already appeared near the fusion line and HAZ. These features indicate that corrosion defects were highly concentrated at the fusion line and HAZ of the girth butt weld. The corrosion hole had completely penetrated the corrosion-resistant liner and extended into the carbon steel backing material. Based on the shape of the corrosion hole, it can be inferred that corrosion initially occurred at the fusion line and HAZ of the root pass in the girth butt weld. This suggests that improper pipe-end seal welding procedures and welding parameters can significantly affect the local chemical composition and microstructure of the HAZ, resulting in a substantial reduction in corrosion resistance.

### 2.2. EDS Analysis

**Figure 3:**
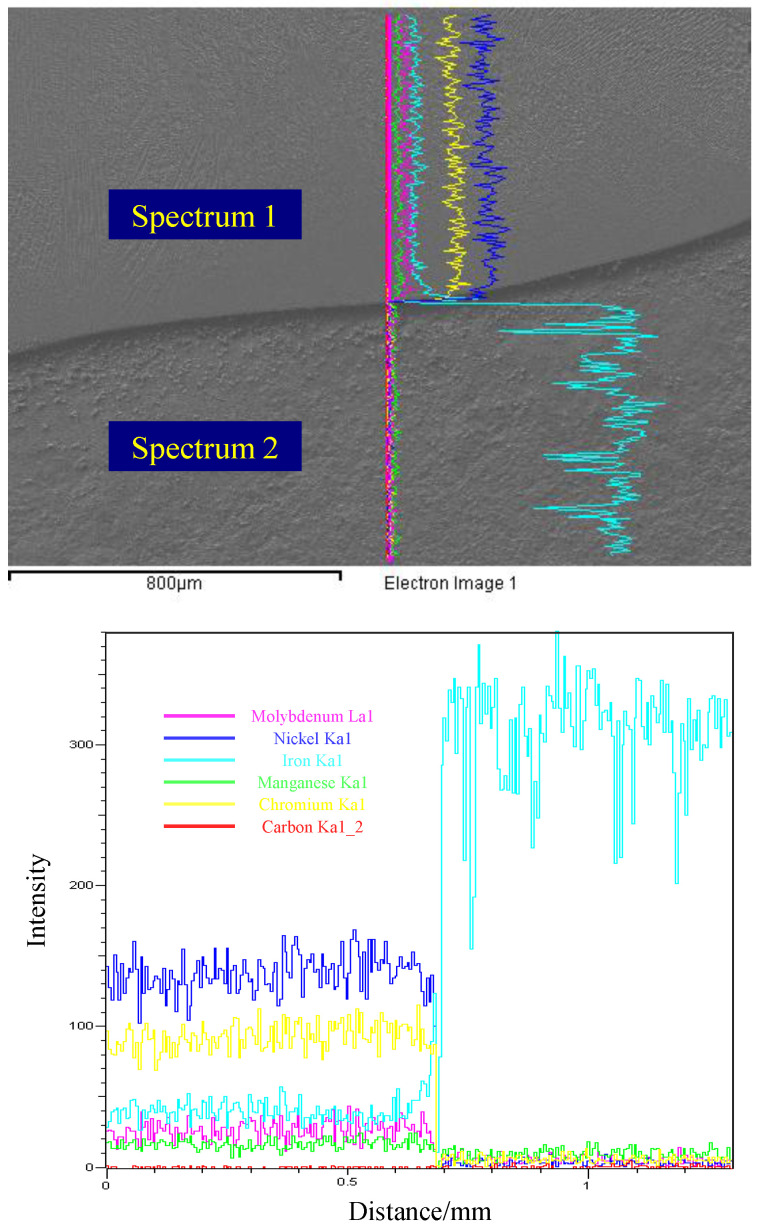
EDS line scan results.

**Figure 4:**
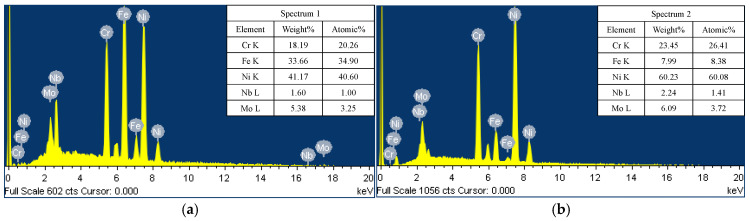
Quantitative microstructural composition analysis (EDS). (**a**) Spectrum 1; (**b**) Spectrum 2.

Based on the EDS line scan and micro-area composition analysis shown in Figure 3 and Figure 4, significant elemental concentration gradients and chemical dilution were observed near the fusion line. Along the line scan path across the fusion line, the contents of key corrosion-resistant elements such as Cr, Ni, and Mo showed a continuous decreasing trend, whereas Fe content increased significantly and remained high on the carbon steel side. This indicates that the welding thermal cycle induced notable elemental diffusion and redistribution between the substrate and the corrosion-resistant layer.

As shown in the figure, at a distance of approximately 0.75 mm from the fusion line, the data presented in Table 1 and Table 2 indicate that the Ni content decreased from 60.23% to 41.17%, Cr from 23.45% to 18.19%, and Mo also exhibited a certain degree of reduction, while Fe increased from 7.99% to 33.66%. These results suggest that under high-heat-input conditions, partial melting of the carbon steel substrate caused diffusion into the upper weld layers, producing a pronounced elemental dilution effect at the fusion line and the adjacent HAZ. This disrupted the original stable distribution of corrosion-resistant alloy elements. Among them, Cr, Ni, and Mo are critical for maintaining the stability of the passive film of the corrosion-resistant alloy. Their locally reduced concentrations may weaken the formation and regeneration of the passive film near the fusion line, increasing susceptibility to pitting, crevice corrosion, and stress corrosion cracking. In addition, high welding heat input may prolong the thermal residence time in the HAZ, further promoting microstructural coarsening, elemental segregation, and local heterogeneity.

However, local elemental dilution does not necessarily lead directly to decreased corrosion resistance, as actual material corrosion behavior is influenced by multiple factors including microstructural evolution, precipitate formation, residual stress state, and service environment. Therefore, elemental dilution and microstructural heterogeneity formed under high-heat-input conditions may increase local corrosion susceptibility in the fusion line and HAZ, making these regions preferential sites for corrosion initiation. Under long-term exposure to corrosive media, these local weak areas may further develop into pitting, localized perforation, or even leakage failure.

In summary, the EDS analysis indicates that welding heat input has a significant effect on elemental distribution near the fusion line, and that the coupling of heat-input-induced elemental dilution, microstructural changes, and residual stress is a key factor contributing to localized corrosion failure in mechanically lined composite pipe welds.

### 2.3. Weld Burn-Through Leakage

To analyze the causes of burn-through leakage in field MLP and reproduce the weld failure behavior, butt welding experiments were designed and carried out in this study. The specific test scheme is shown in Table 3.

**Figure 5:**
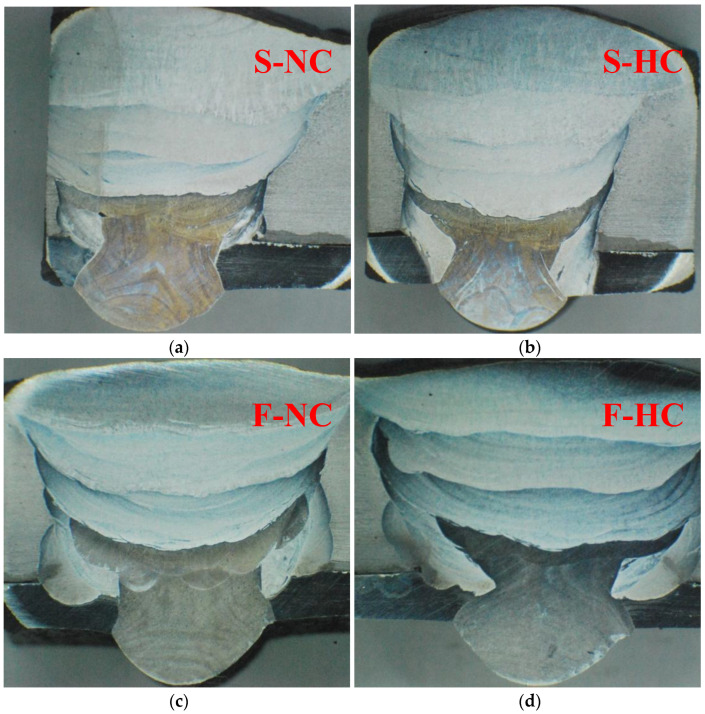
Weld macrostructure. (**a**) solid wire conventional current (**b**) solid wire high current (**c**) Flux-cored wire conventional current (**d**) Flux-cored wire high current.

In all four girth welding experiments conducted in this study, ER309LMo was used as the filler material for the seal welding process. For the girth butt welding process, a multi-layer filler metal combination was adopted, consisting of ER316L for the root pass, ER309LMo for the intermediate transition layers, and CHE507 for the filling and capping layers. This filler metal configuration was designed to ensure both metallurgical compatibility between the carbon steel backing pipe and the corrosion-resistant liner, and satisfactory weld quality. To quantify the influence of different welding process parameters on the HAZ, macro metallographic cross-sections of each specimen were measured (Table 4). The results show that, under solid-wire conditions and normal current (Figure 5a), the HAZ depth below the sealing weld is about 0.507 mm, and the HAZ width at the girth weld is about 1.312 mm. When a higher current is applied (Figure 5b), these values increase to 2.000 mm and 2.247 mm, respectively. For flux-cored wire specimens, under normal current (Figure 5c), the HAZ depth and width are 0.832 mm and 1.425 mm, respectively. Under high current (Figure 5d), they increase to 0.942 mm and 3.395 mm. These dimensional measurements clearly show that higher heat input leads to a significant expansion of the HAZ in both depth and width directions. This inevitably promotes greater dilution of C, Fe, and other harmful elements from the sealing weld into the girth weld. As a result, the chemical composition and microstructure in the fusion line and HAZ are altered, which explains the observed degradation in corrosion resistance.

Based on the above analysis results of the test specimens, obvious corrosion damage was observed at the fusion line and HAZ of MLP girth butt welds. In most cases, the damage had developed into perforation of the liner HAZ, and in some cases further penetrated the entire pipe wall, resulting in burn-through leakage. Examination of the failed welds showed problems such as chemical composition deviation, intergranular corrosion cracking, and corrosion perforation in the related regions. Comprehensive analysis indicates that the weld quality defects were mainly caused by the following factors:

(1) Welding procedure

Excessive welding current during the welding process leads to high heat input, causing local melting of the root weld layer and introducing C, Fe, and other elements into the root pass and regions near the fusion line. This results in dilution of corrosion-resistant alloying elements and reduces local corrosion resistance. At the same time, high heat input produces a significant thermal effect on the stainless steel root pass and hot pass, changing their microstructure and properties. In addition, during carbon steel filling and cap welding, high heat input can easily form regions with high hardness and stress concentration, reducing toughness and crack resistance. Under the combined action of corrosive media and residual stress, rapid cracking is likely to initiate near the fusion line and eventually cause burn-through leakage.

(2) Joint misalignment

Misalignment during MLP butt joint assembly has a significant effect on weld quality. When the misalignment exceeds the effective liner thickness (about 2–2.5 mm), dilution of carbon steel elements into the liner is likely to occur during root welding, resulting in reduced corrosion resistance. In addition, to ensure full penetration during actual welding, the welding speed is often reduced, which further increases heat input, aggravates microstructural degradation, and decreases weld corrosion resistance.

(3) Back shielding protection

When flux-cored wire is used for root welding without effective back shielding gas protection, oxidation is likely to occur in the liner HAZ during welding. This damages the passive film on the stainless steel surface and reduces the effective thickness of the corrosion-resistant layer. Under high-heat-input conditions, the corrosion-resistant alloy layer with a thickness of about 2 mm may even lose its corrosion resistance completely. Corrosion holes observed in the HAZ during intergranular corrosion testing further confirm that insufficient back shielding protection can significantly affect corrosion resistance.

## 3. Finite Element Model

### 3.1. Finite Element Modeling Approach

**Figure 6:**
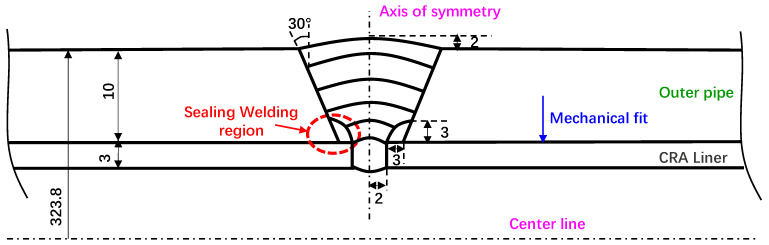
Schematic illustration of the cross-sectional geometry and groove dimensions for the joint.

In this study, a two-dimensional axisymmetric plane strain thermo-mechanical finite element model was established for seal welding of mechanically lined composite pipes. Based on the actual geometry and cross-sectional features of the composite pipe, the outer pipe (base pipe) is a seamless X52 steel pipe with an outer diameter of 323.8 mm and a wall thickness of 10 mm, while the inner corrosion-resistant layer (CRA liner) is made of N08825 with an outer diameter of 303 mm and a wall thickness of 3 mm. The geometric parameters of the composite pipe and the welding sequence are shown in Figure 6.

To replicate the actual field welding process, a single-V groove design was adopted for the pipe ends, with a groove angle of 30° and a root gap of 2 mm. The sealing welding region at the pipe ends and the subsequent girth butt welding region were explicitly defined. Four-node bilinear axisymmetric temperature–displacement coupled elements (CAX4T) were used for the thermo-mechanical analysis. Local mesh refinement was applied in the weld region, fusion line, and adjacent HAZ to ensure the stability and accuracy of the results. The welding heat input was modeled using a moving heat source based on the Goldak double-ellipsoid model. The welding sequence and multi-pass welding procedure were set according to the actual welding process. Displacement constraints were applied as boundary conditions. The interface between the base pipe and the CRA liner was defined as a surface-to-surface contact, with the normal mechanical behavior modeled as hard contact and the tangential behavior simulated using a Coulomb friction model to represent the interlayer relative slip during deformation.

It is worth noting that ER309LMo was uniformly used as the sealing weld material in the finite element model. Since the welding consumables used in this study have similar base chemical compositions and close temperature-dependent mechanical properties, the use of ER309LMo as an equivalent material (Table 5, Table 6 and Table 7) can reasonably represent the thermo-mechanical response during multi-pass welding. This approach ensures the accuracy of both temperature and stress field predictions. At the same time, it simplifies the model and reduces the nonlinear computational cost without affecting the overall evolution of residual stress.

### 3.2. Heat Source Model

**Figure 7:**
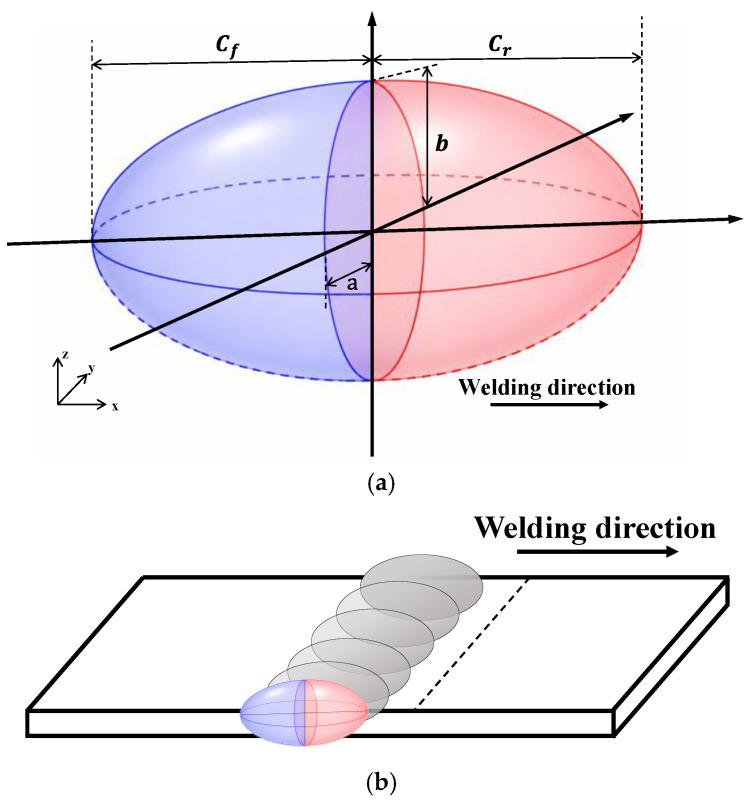
Schematic diagram of double ellipsoidal heat source. (**a**) Schematic diagram; (**b**) moving heat source schematic.

In numerical simulations of thermal processing or welding, the selection of an appropriate heat source model has a significant influence on the subsequent thermo-mechanical response. To accurately describe the spatially non-uniform characteristics of heat input, a double-ellipsoidal heat source model (Figure 7a) is adopted in this study to represent the moving heat source [24]. This model divides the heat source into two half-ellipsoids in space, which represent the energy distribution in the front and rear regions of the heat source, respectively. The volumetric heat flux density can be expressed for the front half-ellipsoid region ($c_{f}\leq x\leq 0$) and the rear half-ellipsoid region ($0\leq x\leq c_{r}$):

(1) $$q_{f}\left(x,y,z\right)=\frac{6\sqrt{3}\cdot f_{f}Q}{\pi abc_{f}}\exp\left(-\frac{3x^{2}}{a^{2}}-\frac{3y^{2}}{b^{2}}-\frac{3z^{2}}{f_{2}^{c}}\right)$$

(2) $$q_{r}\left(x,y,z\right)=\frac{6\sqrt{3}\cdot f_{r}Q}{\pi abc_{r}}exp\left(-\frac{3x^{2}}{a^{2}}-\frac{3y^{2}}{b^{2}}-\frac{3z^{2}}{r_{2}^{c}}\right)$$

Here, $q_{f}$ and $q_{r}$ represent the volumetric heat flux density in the front and rear half-ellipsoids, respectively. Q is the total heat input power, which is generally expressed as Q=ηUI, where η is the thermal efficiency, and U and I are the welding voltage and current, respectively. The parameters a, b, $c_{f}$, and $c_{r}$ are the characteristic semi-axes of the ellipsoid in three directions, which control the spatial distribution range of the heat source. $f_{f}$ and $f_{r}$ are the energy distribution coefficients for the front and rear ellipsoids, respectively, and satisfy the energy conservation condition: $f_{f}+f_{r}=2$.

The parameters are listed in Table 8. The double-ellipsoidal heat source model can represent the asymmetric heat distribution in the front and rear regions during welding, and it is suitable for describing heat concentration in the trailing part of the molten pool. Compared with the conventional Gaussian heat source model, it provides better accuracy in temperature field prediction and weld pool shape simulation. In the numerical implementation, a time-dependent coordinate transformation is introduced to simulate the moving heat source along the welding path, expressed as $x^{\prime }=x-vt$, where v is the heat source traveling speed and t is time. This enables the moving heat source loading along the welding trajectory (Figure 7b). This model provides a reliable heat input condition for temperature field calculation and thermo-mechanical coupled analysis.

## 4. Results and Discussion

### 4.1. Hoop Stress Distribution

**Figure 8:**
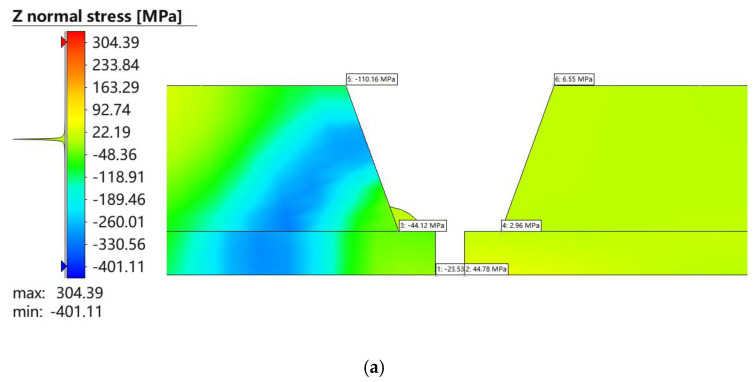

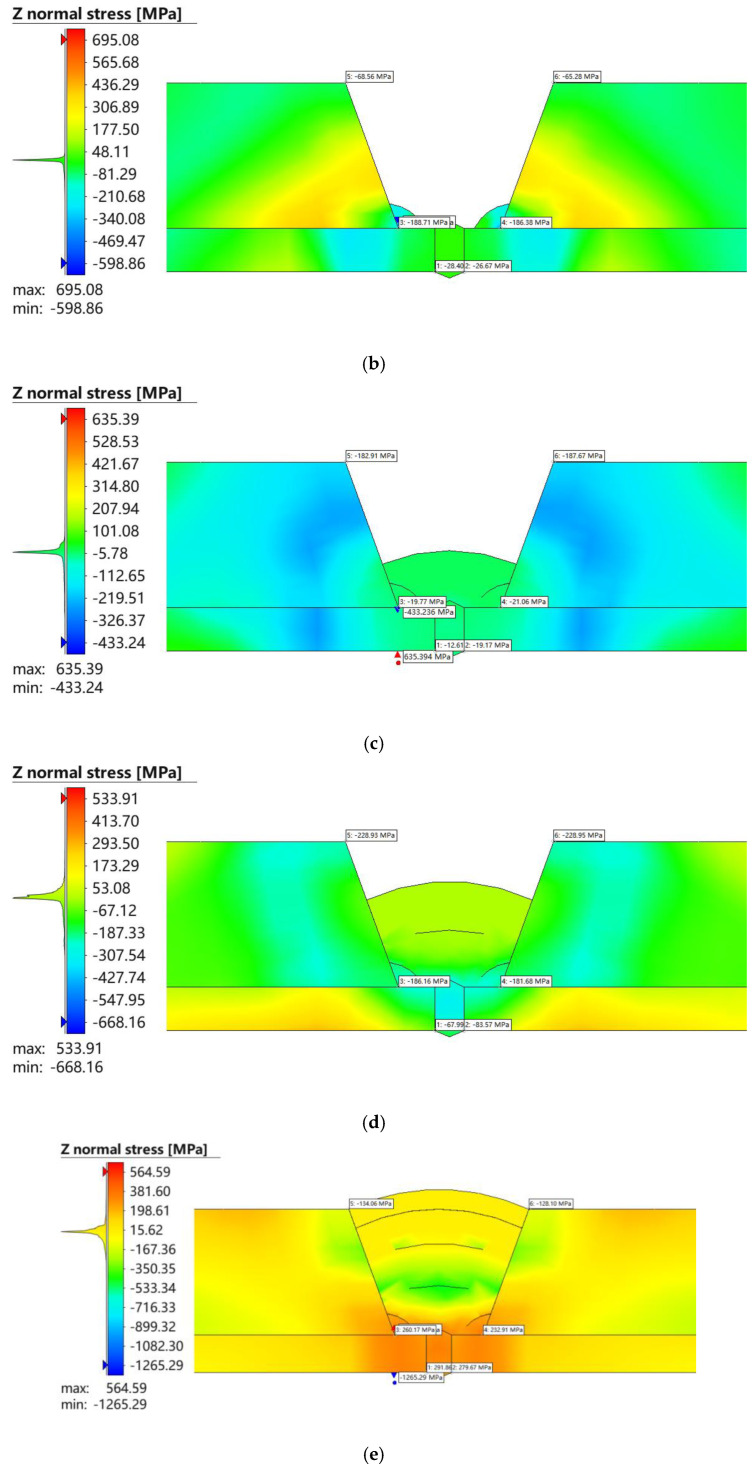
Contour plot of circumferential stress in the cladded layer region. (**a**) First pass of seal welding; (**b**) third pass of seal welding; (**c**) first pass of butt welding; (**d**) second pass of butt welding; (**e**) fourth pass of butt welding.

Based on the aforementioned welding procedure and parameters, the circumferential stress distribution during welding was numerically simulated. Figure 8 shows the Z-direction normal stress distribution in the weld and surrounding material of the lined composite pipe during pipe-end welding, with a stress range from −1265 MPa to 695 MPa. During the first pass of seal welding, the maximum tensile stress was 304.39 MPa, and the maximum compressive stress was −401.11 MPa, with tensile stress concentrated at the weld center and left side, and compressive stress at the bottom and right side. In the third pass of seal welding, tensile stress increased to 695.08 MPa and compressive stress to −598.86 MPa, showing enhanced central tensile stress and increased bottom compressive stress. For the first pass of butt welding, tensile and compressive stresses were 635.39 MPa and −433.24 MPa, respectively, with tensile stress concentrated above the weld and compressive stress at the bottom and edges, indicating a more uniform stress distribution in the weld region. In the second pass, tensile stress decreased to 533.91 MPa, while compressive stress increased to −668.16 MPa. In the fourth pass, tensile and compressive stresses reached 564.59 MPa and −1265.29 MPa, respectively, with compressive stress concentrated at the bottom and liner interface, and tensile stress primarily at the upper center of the weld. Overall, the results indicate high tensile stress at the weld center and concentrated compressive stress at the bottom and edges, with stress forming during the welding thermal cycles and diffusing into the surrounding material.

**Figure 9:**
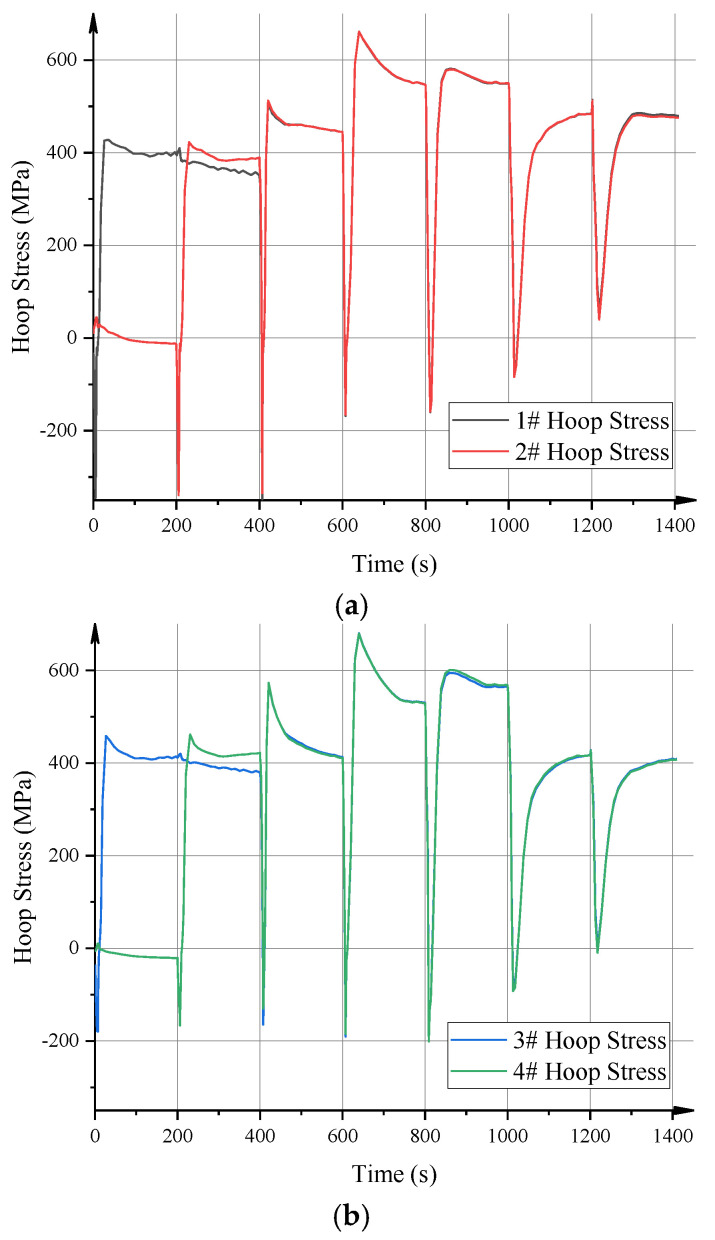

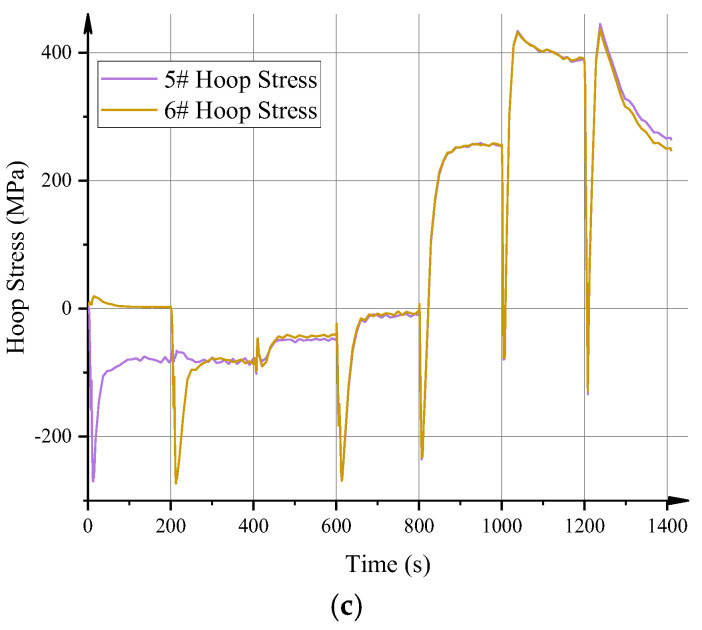
Circumferential stress–time curves at different measurement points. (**a**) Inner surface circumferential stress curve; (**b**) interface circumferential stress curve; (**c**) outer surface circumferential stress curve.

Figure 9 shows the circumferential stress–time curves at different measurement points during the welding of MLP, with all points exhibiting periodic peaks. Measurement points 1# and 2# reached peak stresses of 600–700 MPa and 400–500 MPa, respectively, and rapidly decayed to 0 MPa or slight compressive stress after the welding cycle. Measurement points 3# and 4# reached peaks of 600–700 MPa, with periodic drops of approximately −200 MPa and slight fluctuations over successive cycles, indicating accumulation of circumferential stress in the intermediate region due to thermal cycling. Measurement points 5# and 6# showed peaks of 400–500 MPa, with periodic drops of −150 to −200 MPa; although farther from the weld center, noticeable fluctuations in circumferential stress still occurred during the welding cycles.

**Figure 10:**
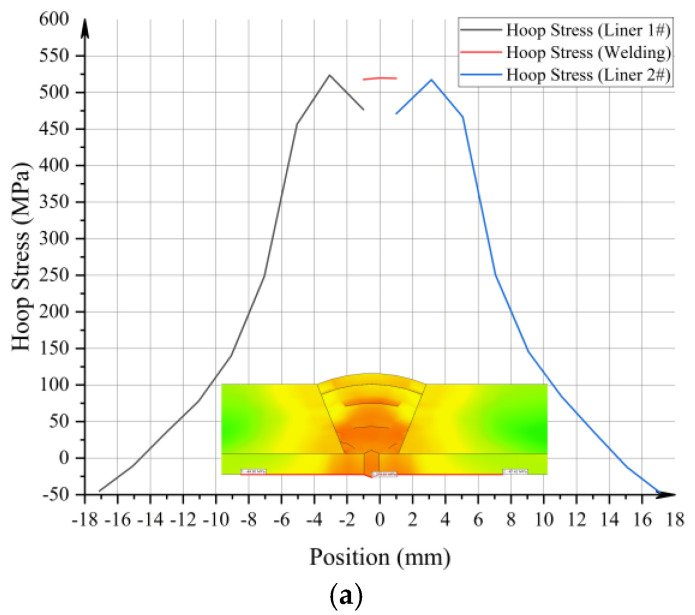

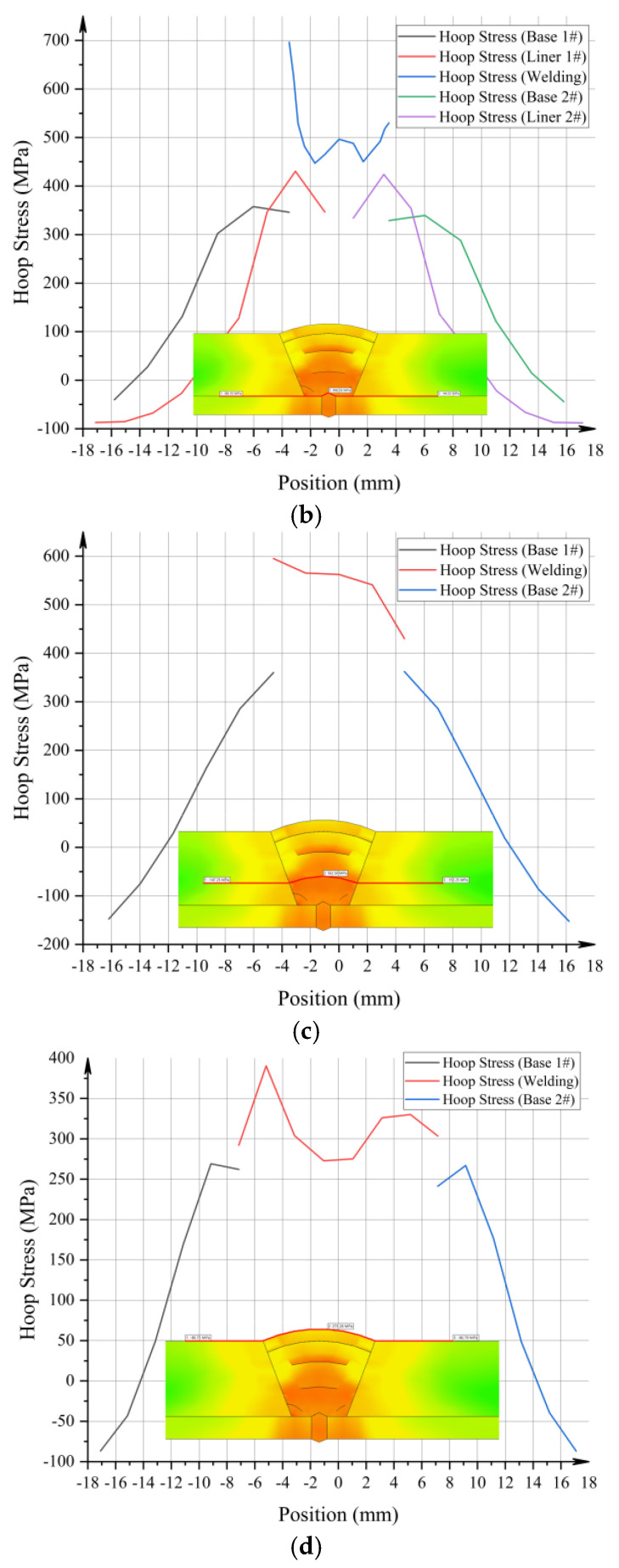
Curves and contour plots of residual circumferential stress after final cooling at various locations. (**a**) Inner surface residual circumferential stress after final cooling; (**b**) interface residual circumferential stress after final cooling; (**c**) mid-Section residual circumferential stress after final cooling; (**d**) outer surface residual circumferential stress after final cooling.

Figure 10 shows the distribution of residual circumferential stress along the cross-section of MLP after welding, for the inner surface, interface, mid-section, and outer surface. In Figure 10a, the peak circumferential stress in the weld region of the inner surface is 500–550 MPa, gradually decreasing to compressive stress toward both sides of the liner. In Figure 10b, at the interface, the residual stress peaks are 300–400 MPa for the base pipe and 400–500 MPa for the liner, with the weld region reaching 600–700 MPa; circumferential stress is observed in both base pipe and liner and decays gradually along the interface. In Figure 10c, at the mid-section, the base pipe residual stress peaks at 300–400 MPa, decreasing to approximately −150 MPa at the sides, while the weld region reaches 550–600 MPa, showing that high tensile stress at the weld center diffuses outward and weakens. In Figure 10d, on the outer surface, the base pipe residual stress peaks at 250–300 MPa, with the weld region at 350–400 MPa, gradually decreasing to compressive stress outward; the outer surface is less affected by the weld thermal cycles, and stress decays noticeably with distance. Overall, the results indicate that residual circumferential stress in the weld region gradually decreases from the inner to the outer surface, providing important data for structural strength assessment.

### 4.2. Axial Stress Distribution

**Figure 11:**
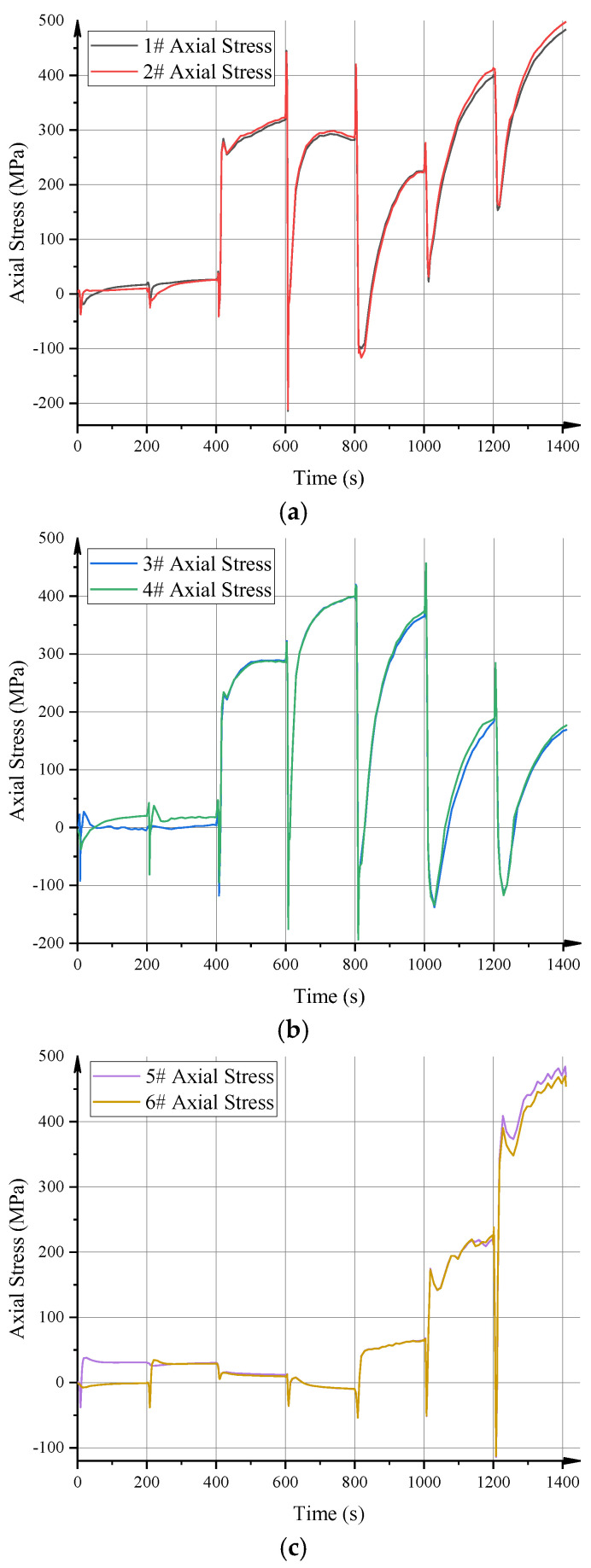
Comparison of axial stress–time curves at different measurement points in the cladded layer. (**a**) Inner surface axial stress curve; (**b**) interface axial stress curve; (**c**) outer surface axial stress curve.

Figure 11 shows the evolution of axial stress over time at different measurement points during multi-pass cladding of MLP, with all points exhibiting periodic peaks corresponding to the welding thermal cycles. Measurement points 1# and 2# reached peak axial stresses of 450–500 MPa, rising rapidly during the welding cycles and decreasing significantly after welding, locally dropping near 0 MPa or briefly into compressive stress, demonstrating a strong thermal cycle response. Measurement points 3# and 4# had peaks of 400–500 MPa, with more gradual variations, indicating cumulative axial stress in the intermediate region under repeated thermal cycles. Measurement points 5# and 6# showed peak stresses of 400–460 MPa, with weak initial response but rapid stress increase under subsequent weld passes, displaying pronounced fluctuations over the welding cycles.

Overall, the axial stress at all points exhibits a typical periodic loading–unloading pattern, with stress peaks synchronized with the welding heat input and dropping during cooling. Stress levels vary with position: points near the weld show higher stresses, while distant points have lower amplitudes, yet stresses gradually accumulate under thermal cycling, leading to substantial residual tensile stress.

**Figure 12:**
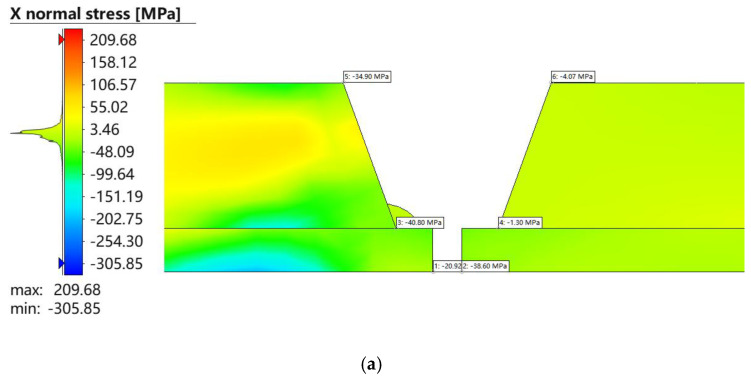

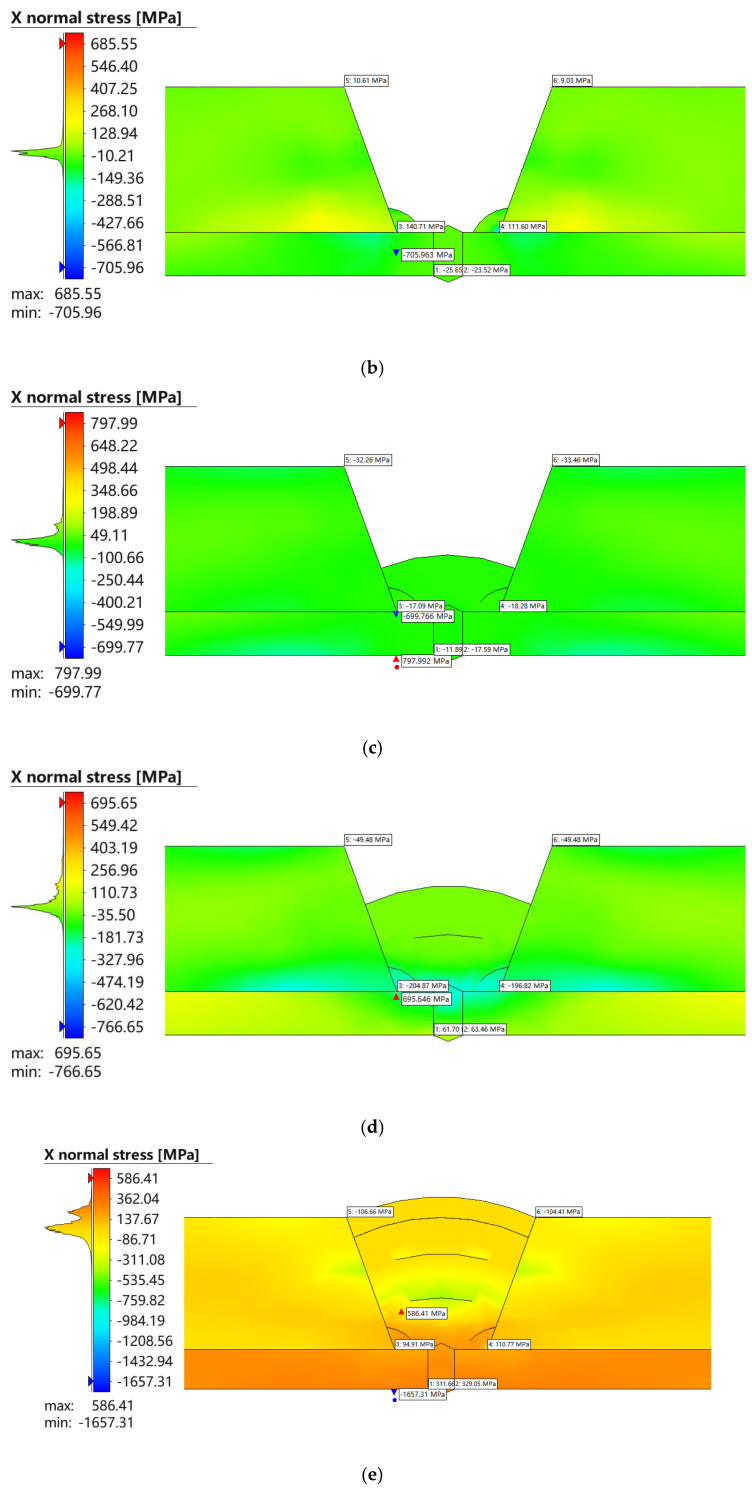
Evolution of X-direction normal stress distribution during different stages of welding. (**a**) First pass of seal welding; (**b**) third pass of seal welding; (**c**) first pass of butt welding; (**d**) second pass of butt welding; (**e**) fourth pass of butt welding.

Figure 12 shows the evolution of X-direction normal stress at different stages during the pipe-end welding of MLP, with the overall stress range from −1657 MPa to 798 MPa, indicating pronounced thermal cycle-driven behavior. In Figure 12a, during the first pass of seal welding, the maximum tensile stress was 209.68 MPa and the maximum compressive stress was −305.85 MPa, with tensile stress mainly distributed at the upper part of the weld and the bevel sidewall, and compressive stress concentrated at the bottom and near the liner interface. In Figure 12b, during the third pass of seal welding, tensile and compressive stresses reached 685.55 MPa and −705.96 MPa, respectively, with tensile stress concentrated at the weld center and spreading toward both sides. In Figure 12c, for the first pass of butt welding, the maximum tensile stress was 797.99 MPa and compressive stress −699.77 MPa; the weld center showed enhanced tensile stress with a clear concentration zone, while the bottom compressive stress region expanded. In Figure 12d, in the second pass of butt welding, tensile and compressive stresses were 695.65 MPa and −766.65 MPa, respectively, with increased compressive stress at the bottom and liner interface, a significantly enlarged compressive region, and heat conduction downward producing stronger restraint at the interface. In Figure 12e, in the fourth pass, maximum tensile and compressive stresses were 586.41 MPa and −1657.31 MPa, respectively, showing strong compressive stress concentration at the bottom and interface, while tensile stress was mainly at the upper center of the weld and relatively reduced.

Overall, high tensile stress zones persist at the weld center, while high compressive stresses gradually accumulate at the bottom and liner interface. With increasing weld passes, stress spreads from localized concentrations to surrounding areas and evolves cumulatively under thermal cycling.

**Figure 13:**
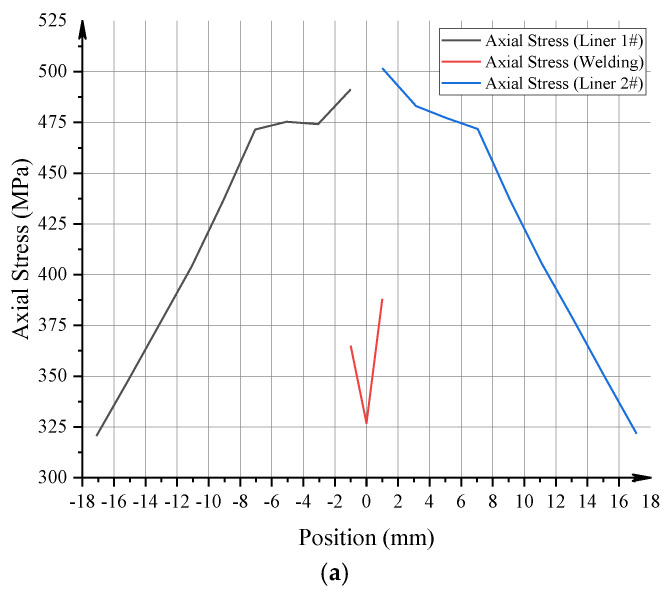

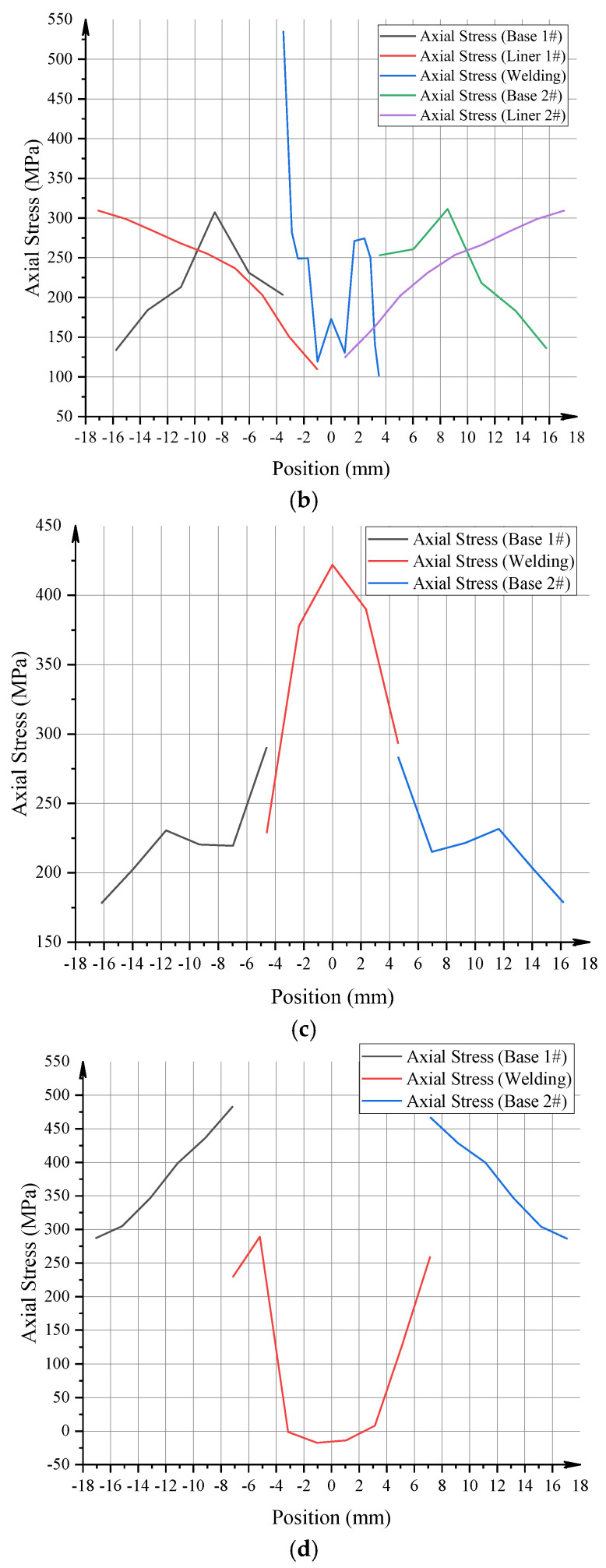
Residual axial stress curves at different interfaces. (**a**) Inner surface residual axial stress after final cooling; (**b**) interface residual axial stress after final cooling; (**c**) mid-section residual axial stress after final cooling; (**d**) outer surface residual axial stress after final cooling.

Figure 13 shows the distribution of axial residual stress along the cross-section of MLP after welding, for the inner surface, interface, mid-section, and outer surface, with distinct stress levels and trends in each region. In Figure 13a, the peak axial stress in the weld region of the inner surface is 480–510 MPa, forming a clear high tensile stress concentration at the weld center. Stress gradually decreases along both sides of the liner, dropping to 300–350 MPa in regions far from the weld. The inner surface is directly affected by the welding heat input, exhibiting the highest and most concentrated stress. In Figure 13b, at the interface, axial stress peaks are 300–350 MPa on the base pipe side and 280–320 MPa on the liner side, while local stress fluctuations in the weld region are significant, with peaks exceeding 500 MPa. Near the weld, stress variations are evident and the distribution is non-uniform. In Figure 13c, at the mid-section, the base pipe axial stress peaks at 280–300 MPa, gradually decreasing to 180–220 MPa at the sides. Stress in the weld region increases significantly, with peaks of 400–420 MPa, gradually weakening outward from the weld center. In Figure 13d, on the outer surface, axial stress in the base pipe ranges from 280 to 480 MPa. Local stress relief occurs in the weld region, with minimum values around −20 MPa, followed by stress distribution of 250–300 MPa on both sides. The outer surface exhibits a relatively mild stress gradient and is less affected by the welding thermal cycles.

Overall, high axial residual stress zones form in the weld region and gradually decrease from the inner to the outer surface, with the interface and mid-section showing clear stress transitions and fluctuations.

### 4.3. Equivalent Stress Distribution

**Figure 14:**
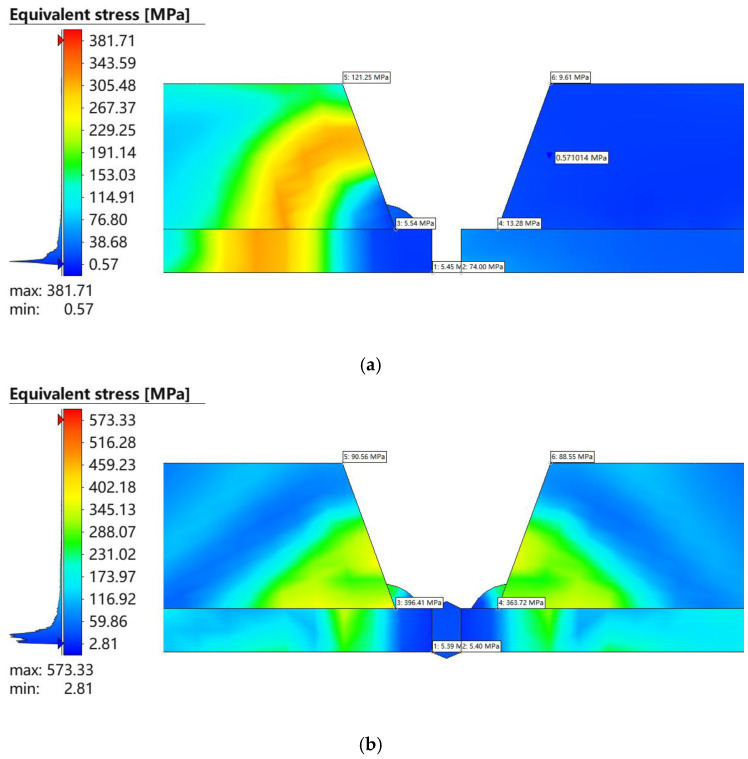

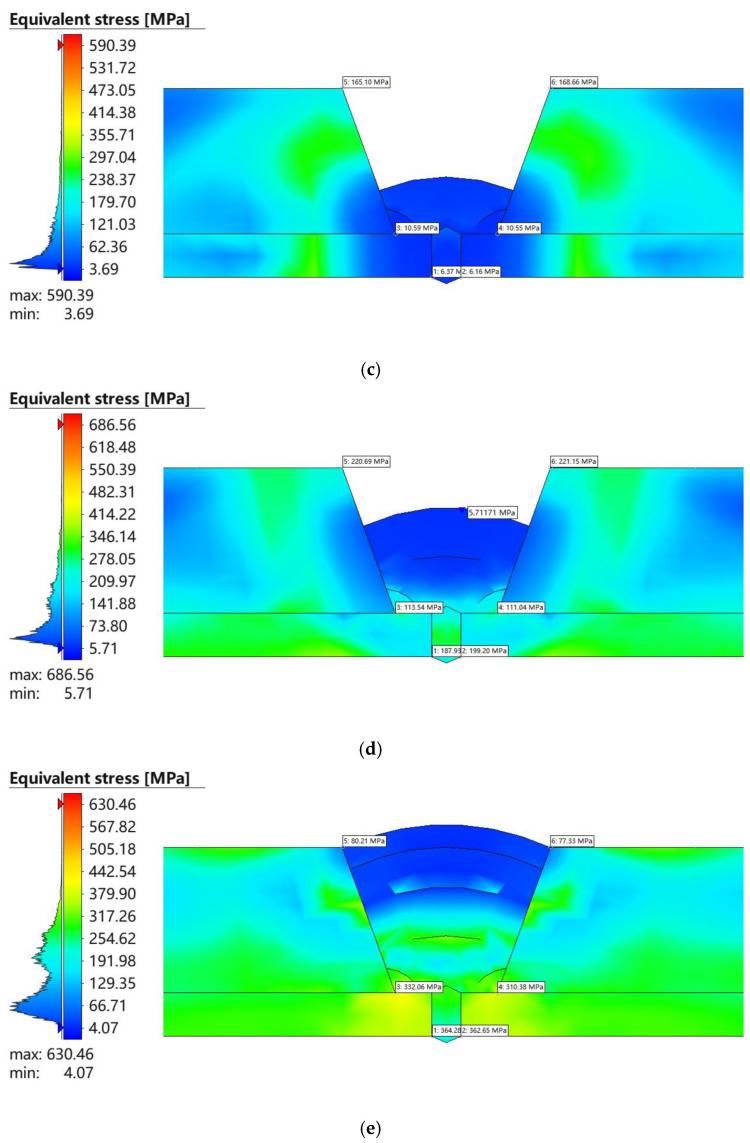
Evolution of equivalent stress distribution in the cross-section of the composite pipe at various welding stages. (**a**) First pass of seal welding; (**b**) third pass of seal welding; (**c**) first pass of butt welding; (**d**) second pass of butt welding; (**e**) fourth pass of butt welding.

Figure 14 shows the evolution of equivalent stress across the cross-section of MLP at different welding stages. The equivalent stress increases progressively during the weld filling process, with the maximum value rising from 381.71 MPa in the first pass of seal welding to 686.56 MPa in the second pass of butt welding, and slightly decreasing to 630.46 MPa in the fourth pass of butt welding. This indicates that under the combined effects of welding thermal cycles and material constraints, stress continuously accumulates at the groove root and interface. In Figure 14a, during the first pass of seal welding, equivalent stress ranges from 0.57 to 381.71 MPa, concentrated mainly at the local groove area and exhibiting noticeable asymmetry. In Figure 14b, for the third pass of seal welding, the range increases to 2.81–573.33 MPa, with high-stress regions extending from the groove root toward the base pipe; as the number of passes increases, stress concentration in the and interface becomes more pronounced. In Figure 14c, during the first pass of butt welding, equivalent stress ranges from 3.69 to 590.39 MPa, with reduced stress at the groove root and high-stress regions extending into the base pipe. In Figure 14d, during the second pass of butt welding, the range is 5.71–686.56 MPa, with high-stress regions enhanced on both sides of the groove and near the base–liner interface. This stage exhibits the strongest combined effect of thermal input accumulation and structural constraint, representing a critical stage for equivalent stress concentration. In Figure 14e, during the fourth pass of butt welding, equivalent stress ranges from 4.07 to 630.46 MPa, with stress extending from the upper groove toward the base–liner interface, where interface stress increases to 364.28 MPa, forming the primary stress concentration zone. The results indicate that the groove root and base–liner interface are the regions of most significant stress accumulation and should be the focus of residual stress control and structural integrity assessment for bimetallic composite pipe welding.

**Figure 15:**
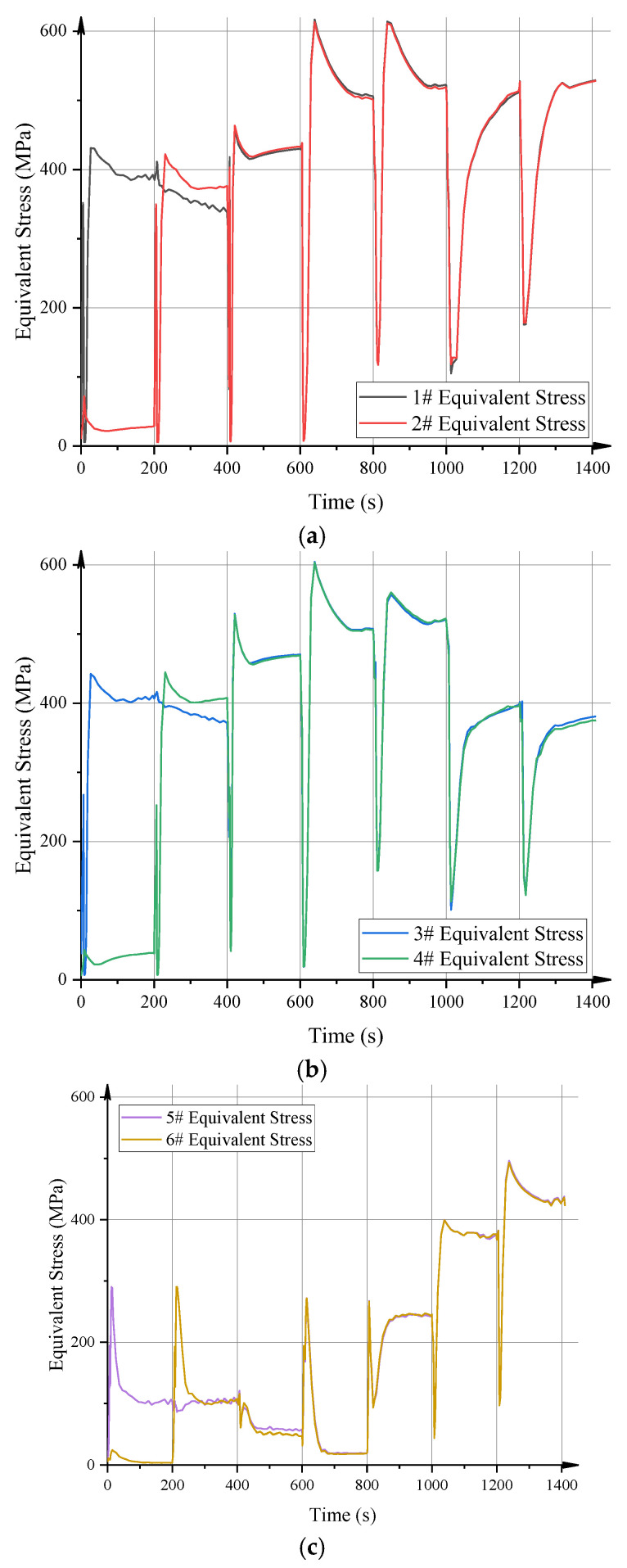
Comparison of equivalent stress–time curves at different measurement points in the cladded layer. (**a**) Inner surface equivalent stress curve; (**b**) interface equivalent stress curve; (**c**) outer surface equivalent stress curve.

Figure 15 shows the evolution of equivalent stress over time at six measurement points during the welding of MLP, with all points exhibiting pronounced periodic behavior corresponding to the multi-pass welding thermal cycles. Measurement points 1# and 2# experienced the highest overall stress levels, with peaks exceeding 600 MPa. Specifically, point 1# maintained 380–420 MPa during the initial stage, while point 2# reached peak values of 600–620 MPa at approximately 620 s and 830 s, stabilizing around 520 MPa after welding. Measurement points 3# and 4# had peak equivalent stresses of 600–650 MPa. Point 3# maintained a high stress level of 500–550 MPa between 830 and 1000 s, whereas point 4# increased gradually in the early stage and stabilized at 360–400 MPa in the later stage. Measurement points 5# and 6# exhibited low stress initially, being farther from the heat source and less affected, but after 800 s, stress rapidly accumulated, with peak values reaching 450–500 MPa and eventually stabilizing at 420–440 MPa. These results indicate that equivalent stress gradually accumulates with successive weld passes. Measurement points near the weld and respond earlier and reach higher peaks, while points farther from the weld show delayed response. Multi-pass thermal cycles induce repeated local stress relaxation and reloading, ultimately forming a high residual equivalent stress state dominated by tensile stress.

**Figure 16:**
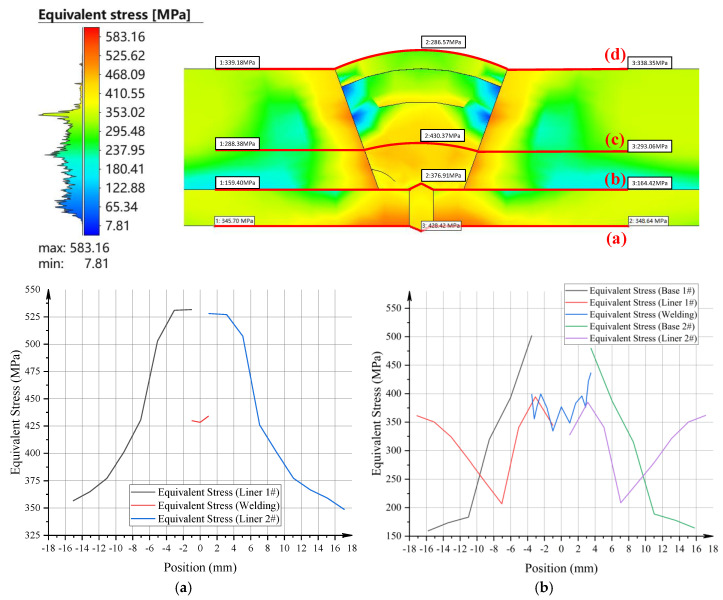

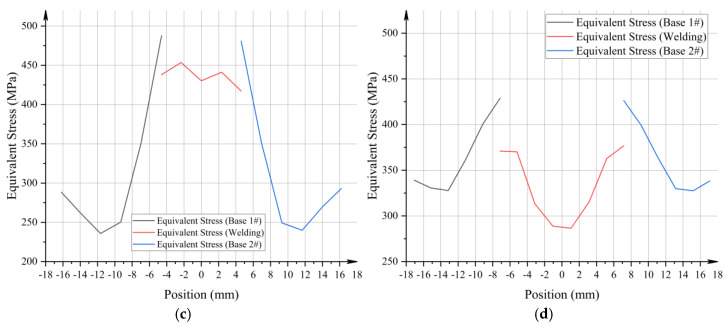
Residual equivalent stress distributions on different surfaces after final cooling: (**a**) inner surface, (**b**) interface, (**c**) mid-thickness section, and (**d**) outer surface.

Figure 16 shows the distribution of residual stress along the thickness at different cross-sectional positions of MLP after welding, exhibiting a general trend of gradual decay from the weld region toward both sides. In Figure 16a, at the inner surface, the equivalent stress in the weld region peaks at 500–550 MPa, forming a pronounced high-stress concentration at the weld center. Stress gradually decreases to approximately 350 MPa along both sides of the liner. The inner surface is directly affected by the welding heat input, showing the most significant stress concentration. In Figure 16b, at the interface, the peak equivalent stress is 500–520 MPa on the base pipe side and approximately 380–400 MPa on the liner side, with local peaks in the weld region reaching 430–450 MPa. The stress gradient near the interface is steep, gradually decaying along the interface toward both sides. In Figure 16c, the equivalent stress in the welded region of the mid-thickness section reaches a peak value of 400–460 MPa, while the base pipe region peaks at 480–500 MPa, gradually decreasing outward from the weld center. In Figure 16d, at the outer surface, stress levels are overall lowest, with weld region peaks of 275–350 MPa, base pipe peaks of 425–450 MPa, and gradual reduction toward regions farther from the weld. Compared with the inner surface, the outer surface is less affected by welding thermal cycles, exhibiting a milder stress gradient. Overall, residual equivalent stress forms peak concentrations in the weld region, gradually decreasing from the inner to outer surfaces along the thickness and decaying along the cross-section toward both sides. The weld and its adjacent areas are critical regions for residual stress control and structural integrity assessment.

### 4.4. Results and Analysis

In summary, significant residual stress accumulation and uneven stress distribution were observed during the pipe-end welding of mechanically lined composite pipes. The hoop residual stress reaches its peak in the weld center and near the HAZ, with a maximum tensile hoop stress of 500–550 MPa on the inner surface and an axial residual stress peak of 480–510 MPa. As the number of welding passes increases, the stress gradually extends toward the groove root and the interface between the base pipe and the liner, forming regions of high stress concentration.

Equivalent stress analysis indicates that stress accumulation is most pronounced at the groove root and the base–liner interface during multi-pass welding. The maximum equivalent stress reaches 686.56 MPa at the second pass of the girth weld, followed by a slight decrease, yet remains at a high level. This suggests that these regions are critical for residual stress control and structural integrity assessment. Moreover, areas with high tensile residual stress concentration are also susceptible to crack initiation and propagation. The stress gradient at the interface is related to the risk of interfacial failure, while the coupling effect of inner-surface stress and thermal cycles may promote the initiation of localized corrosion and pitting.

Table 9 summarizes the hoop, axial, and equivalent stress values for different weld passes, while Table 10 presents the peak hoop, axial, and equivalent stresses at different measurement points and interfaces.

Based on microstructural observations and EDS analysis, the welding heat input induces diffusion of elements from the carbon steel substrate into the corrosion-resistant liner. Near the fusion line, a pronounced Fe enrichment and a reduction in corrosion-resistant elements such as Cr, Ni, and Mo are observed, exhibiting typical chemical dilution and interfacial elemental redistribution. The overlap of these local compositional changes with regions of high tensile residual stress provides favorable conditions for pitting and localized perforation, revealing the mechanism of localized failure in the weld joint. Further analysis indicates that the combined effects of HAZ expansion, elemental dilution, and residual stress contribute to the formation of a weakly corrosion-resistant zone in the weld and its adjacent areas. Through mechanisms such as stress corrosion, this may lead to penetrative corrosion. The results demonstrate that excessive heat input simultaneously causes HAZ expansion, elemental dilution, and high residual stress concentration, thereby reducing the local corrosion resistance of the joint and increasing the risk of failure.

To further evaluate the validity of the thermo-mechanical finite element model developed in this study, the simulated residual stress distributions were compared with numerical and experimental results reported in previous studies [25,26,27] on mechanically lined composite pipe welding. The comparison indicates that the residual stress features and high-stress concentration regions obtained in this study are generally consistent with the findings of previous research. Obeid et al. [28] reported through numerical simulations of the welding process in mechanically lined composite pipes that the maximum tensile residual stresses were primarily concentrated near the weld root and the liner–base pipe interface, with peak values ranging from approximately 450 to 650 MPa. Dong et al. [29] in their study of girth-welded 2205/X65 bimetallic pipes, found that the peak tensile residual stresses in the weld and HAZ were approximately 450–600 MPa, with higher stress levels on the inner surface than on the outer surface. In the present study, the simulated peak circumferential residual stress on the inner surface was 500–550 MPa, while the peak axial residual stress ranged from 480 to 510 MPa. The maximum equivalent stress at the interface reached 686.56 MPa. Both the locations of stress concentration and the stress magnitude ranges are in good agreement with the aforementioned studies. These results demonstrate that the present model can reasonably capture the thermo-mechanical response and residual stress evolution during the welding process of mechanically lined composite pipes, thereby validating the reliability of the model predictions.

In the present study, the residual hoop and axial stresses in the weld region are primarily concentrated in the weld center and the adjacent HAZ, gradually decaying along the thickness from the inner to the outer surface. In addition, significant stress concentrations and gradient variations are observed near the base–liner interface. These findings are in good agreement with the reported residual stress evolution in previous studies. Moreover, the peak equivalent stress values obtained are comparable to those reported for similar welded structures in the literature, indicating that the current finite element model can reasonably capture the thermo-mechanical response of mechanically lined composite pipes during welding.

## 5. Conclusions

(1) The EDS analysis and metallographic observations indicate that welding heat input promotes elemental diffusion and metallurgical dilution from the carbon steel substrate toward the corrosion-resistant liner, resulting in significant compositional redistribution near the fusion line. The Fe content increased to 33.66 wt.%, while the concentrations of key corrosion-resistant elements such as Cr and Ni gradually decreased, exhibiting typical interfacial elemental dilution characteristics. These compositional changes may weaken the stability of the passive film near the fusion line and increase susceptibility to localized corrosion.
(2) The width of the HAZ increases significantly with increasing welding heat input. Under high-current welding conditions, the HAZ width of the solid-wire girth weld increased from 1.312 mm to 2.247 mm. Excessive welding current, low welding speed, joint misalignment, and insufficient backside shielding gas may lead to local overheating, thinning of the corrosion-resistant layer, and microstructural degradation, thereby increasing the risk of localized corrosion and premature failure of welded joints.
(3) The finite element results show that significant residual tensile stress concentrations tend to develop in the weld region and adjacent HAZ during multi-pass welding. The peak residual hoop stress on the inner surface ranges from 500 to 550 MPa, while the peak residual axial stress ranges from 480 to 510 MPa. During the girth weld filling stage, the maximum equivalent stress near the interface between the outer pipe and the liner reaches 686.56 MPa. The predicted residual stress distributions are generally consistent with previously reported results for mechanically lined composite pipes, indicating that the present model can reasonably capture the thermo-mechanical evolution during the welding process.
(4) This study primarily investigated elemental dilution, microstructural evolution, and residual stress distribution during pipe-end welding of mechanically lined composite pipes. Future work should focus on further optimization of welding parameters, including welding current, voltage, welding speed, interpass temperature, and shielding gas conditions, to establish a more stable welding procedure. In addition, multi-field coupled analyses involving residual stress, mechanical loading, and corrosion damage under realistic service conditions, such as internal pressure, external bending moment, and corrosive environments, should be conducted. Combined with electrochemical corrosion experiments and residual stress measurements, these studies would enable a more comprehensive understanding of the long-term service failure behavior of welded joints and provide valuable guidance for structural integrity assessment and engineering applications of bimetallic composite pipe joints.

## Figures and Tables

**Table 1:** Elemental composition of corrosion products (wt.%).

Element	Cr K	Fe K	Ni K	Nb L	Mo L
Spectrum 1	18.19	33.66	41.17	1.60	5.38
Spectrum 2	23.45	7.99	60.23	2.24	6.09

**Table 2:** Elemental composition of corrosion products (At%).

Element	Cr K	Fe K	Ni K	Nb L	Mo L
Spectrum 1	20.26	34.90	40.60	1.00	3.25
Spectrum 2	26.41	8.38	60.08	1.41	3.72

**Table 3:** Welding Procedure.

Specimen ID	Filler Wire Type	Current (A)	Voltage (V)	Welding Speed (mm/s)	Wire Diameter (mm)	Interpass Temperature (°C)	Shielding Gas	Remarks
S-NC	Solid Wire	160	24	5	2.4	120	Ar	Conventional Current
S-HC	Solid Wire	250	26	4	2.4	150	Ar	High Current
F-NC	Flux-Cored Wire	180	24	5	1.6	120	CO_2_	Conventional Current
F-HC	Flux-Cored Wire	260	26	4	1.6	150	CO_2_	High Current

**Table 4:** Measured HAZ Dimensions.

Sample	HAZ Depth (Seal Weld)	HAZ Width (Girth Weld)
S-NC	0.507 mm	1.312 mm
S-HC	2.000 mm	2.247 mm
F-NC	0.832 mm	1.425 mm
F-HC	0.942 mm	3.395 mm

**Table 5:** Thermo-mechanical properties of X52.

Temperature\°C	Young’s Modulus\10^5^·MPa	Poisson’s Ratio	Yield Stress\MPa	Thermal Expansion\×10^−5^·°C^−1^	Specific Heat\J/kg·°C	Conductivity\W·m^−1^·K^−1^
20	2.06	0.30	360.50	1.14	461	50.20
200	1.93	0.30	315.40	1.26	478	47.90
400	1.81	0.31	268.20	1.39	525	43.10
600	1.66	0.31	175.60	1.49	595	36.40
800	1.48	0.31	95.30	1.61	645	28.80
1000	1.32	0.32	72.10	1.75	685	30.50
1200	1.05	0.32	45.80	1.92	665	32.80
1500	0.85	0.33	18.50	2.08	655	34.60
2000	0.65	0.34	11.20	2.45	810	15.20

**Table 6:** Thermo-mechanical properties of N08825.

Temperature\°C	Young’s Modulus\10^5^ MPa	Poisson’s Ratio	Yield Stress\MPa	Thermal Expansion\×10^−5^·°C^−1^	Specific Heat \J/kg·°C	Conductivity\W·m^−1^·K^−1^
20	1.97	0.30	275	1.35	406.52	54
250	1.84	0.30	240	1.45	422.2	49
500	1.71	0.31	201	1.53	505.8	42
750	1.58	0.31	89	1.61	549.8	29
1000	1.45	0.32	67	1.69	593.75	32
1250	1.31	0.32	40	1.77	582.8	34.5
1500	1.18	0.33	13	1.85	571.76	37
1750	1.05	0.33	11	1.93	646.6	26.2
2000	0.92	0.34	9	2.01	721.3	15.4

**Table 7:** Thermo-mechanical properties of ER309LMo.

Temperature\°C	Young’s Modulus\10^5^ MPa	Poisson’s Ratio	Yield Stress\MPa	Thermal Expansion\×10^−5^·°C^−1^	Specific Heat\J/kg·°C	Conductivity\W·m^−1^·K^−1^
20	1.95	0.30	385	1.50	450	14.5
300	1.76	0.30	265	1.65	520	19
550	1.58	0.31	195	1.77	570	23.4
850	1.30	0.31	110	1.91	630	28.5
1150	0.97	0.32	44	2.02	678	33.1
1400	0.43	0.33	18	2.09	726	36.6
1650	0.09	0.34	7	2.16	780	39.2
1850	0.03	0.35	3	2.21	820	40.8
2000	0.01	0.35	2	2.25	850	42

**Table 8:** Specific parameters of double-ellipsoidal heat source.

Parameter	Front Length $C_{f}$/mm	Rear Length $C_{r}$/mm	Width a/mm	Depth b/mm	Gaussian Parameter M
Sealing welding	1.85	5.82	2.65	2.38	3.0
Girth welding	2.77	8.45	4.36	3.65	3.0

**Table 9:** Hoop, axial, and equivalent stress values for different weld passes.

		Hoop Stress		Axial Stress		Equivalent Stress
		Maximum Tensile Stress/MPa	Maximum Compressive Stress/MPa	Maximum Tensile Stress/MPa	Maximum Compressive Stress/MPa	Equivalent Stress Range/MPa
Seal Welding	Pass 1	304.39	−401.11	209.68	−305.85	0.57–381.71
	Pass 3	695.08	−598.86	685.55	−705.96	2.81–573.33
Girth Welding	Pass 1	635.39	−433.24	797.99	−699.77	3.69–590.39
	Pass 2	533.91	−668.16	695.65	−766.65	5.71–686.56
	Pass 4	564.59	−1265.29	584.41	−1657.31	4.07–630.46

**Table 10:** Peak hoop, axial, and equivalent stress ranges at different measurement points and interfaces.

	Different Measurement Points			Different Interfaces			
	1#, 2#	3#, 4#	5#, 6#	Inner Surface	Interface	Mid-Thickness Section	Outer Surface
Peak Hoop Stress (MPa)	600–700	600–700	400–500	500–550	600–700	550–600	350–400
Peak Axial Stress (MPa)	450–500	400–500	400–460	480–510	500–550	400–420	250–300
Peak Equivalent Stress (MPa)	600–620	600–650	450–500	500–550	430–450	400–460	275–350

## Data Availability

The original contributions presented in this study are included in the article. Further inquiries can be directed to the corresponding authors.
